# Stapes surgery in Sweden: evaluation of a national-based register

**DOI:** 10.1007/s00405-017-4510-2

**Published:** 2017-03-11

**Authors:** Karin Strömbäck, Lars Lundman, Andreas Bjorsne, Joakim Grendin, Anna Stjernquist-Desatnik, Ylva Dahlin-Redfors

**Affiliations:** 1 Departments of Otorhinolaryngology, Uppsala University Hospital, 75185 Uppsala, Sweden; 2 Karlstad Hospital, Karlstad, Sweden; 3 Sahlgrenska Tech Audiologist University Hospital, Gothenburg, Sweden; 4 Östersund Hospital, Östersund, Sweden; 5 Lund University Hospital, Lund, Sweden; 6 Sahlgrenska University Hospital, Gothenburg, Sweden

**Keywords:** Otosclerosis, Surgical register, Quality indicators

## Abstract

The aim of the National Quality Registries is to monitor the outcome of healthcare given to patients. The Swedish Quality register for otosclerosis surgery is one of the nine official national registers for ear, nose and throat diseases in Sweden. Since 2004, surgical and audiological results and patient satisfaction scores have been systematically collected from a majority of the ear, nose and throat clinics performing stapes surgery in Sweden. The results of 1688 patients who underwent primary operations for otosclerosis were evaluated for 24 out of totally 26 clinics performing stapes surgery, between 2004 and 2010. The most common surgical technique reported was stapedotomy accomplished in an overnight stay. A majority of patients experienced improved hearing, and were satisfied with the preoperative counselling. Successful surgery, defined as an ABG closure ≤10 dB HL, was achieved in 69%, improvement in AC by ≥20 dB in 63% and BC not worsened by more than ≥5 dB in 93% of the patients. An overall low incidence of postoperative complications was reported. The outcome for ABG and BC was demonstrated to be independent of the number of operations performed by each clinic. An evaluation of the register and the results from the SQOS revealed that stapes surgery is a safe procedure with good hearing outcomes, low complication rates and a high rate of patient’s satisfaction on a national level.

## Introduction

Today 134 National Swedish Quality registries are providing useful data, which can be used to improve the quality of medical care in many different medical fields. The registries, which are obtaining central fundings, contain valuable data for both individual patients and larger groups of patients with similar diagnosis. Consequently, the plethora of different predisposing factors and treatment outcome can be scientific evaluated and compared in many different ways. All data are annually monitored and approved by an executive committee.

Since 1997, an ear, nose and throat (ENT) national-based register has been developed. The Swedish quality register for otosclerosis surgery (SQOS) is one of the nine national registers for ENT diseases and is independent of the National Institute of Health Care (NIHC). Each year, a comparison between the NIHC and the SQOS is performed. All stapes surgery is registered with the NIHC and 80% of the surgical procedures are reported by the clinics in the SQOS.

The overall goal of the SQOS, which was established in 2004, was to enable the comparisons of surgical results from different clinics to improve and secure the quality of stapes surgery. The aim was to identify all the patients undergoing primary and revision stapes surgeries and to create a comprehensive national database. In contrast to the NIHC where only the numbers of surgical procedures are registered, the surgical complications, audiometric results, postoperative complications and patient satisfaction are assessed in the SQOS.

The present study is the first to evaluate the SQOS and describe the surgical technique, audiological results and preoperative patients’ information, in patients undergoing primary surgery for otosclerosis. Another goal was to investigate if a larger numbers of operations in a clinic had a positive influence on the outcomes.

Otosclerosis is considered a multifactorial disorder in which genetic inheritance and autoimmune factors are important features for developing the disease [1]. Otosclerosis is the most common cause of conductive hearing impairment in young adults and is estimated to affect 0.3% of the population [2]. It is almost twice as frequent in women and is more common in middle-aged individuals. The hearing loss is mainly conductive, but in a majority of cases, sensorineural hearing loss of varying degrees develops without association to age related hearing loss [3–5]. Otosclerosis is treated with hearing aids and/or stapes surgery. In Sweden, which has a population of 10 million people, an average of 450 otosclerosis ears are operated on annually, with the number varying between 380 and 580 during the last 10 years.

## Materials and methods

The regional register centers and steering committee (registry organization) runs and develops the registry and is responsible for the professional and ethical anchoring of the registry. It is approved by the ethical review board and processing of the data can be performed at individual level. Every year, the results are analyzed and selected data presented in a public presentation from the Swedish County Council to show the possible improvement in health care.

The results of the primary surgical treatment (stapedotomy, stapedectomy) of adult otosclerosis patients (>15 years of age) were assessed and evaluated. The exclusion criteria were tympanosclerosis, osteogenesis imperfecta and juvenile stapes fixation.

Three separate documents were filled out by the surgeon, while a questionnaire was filled out by the patient. The documents were then registered in the database.

Document 1 covered preoperative data concerning age, sex, audiometry and possible previous otosclerosis surgeries.

Document 2 included the surgical technique performed (stapedotomy, stapedectomy, prosthesis, drill or laser), anaesthesia (local, general) and postoperative nursing (outpatient-surgery, overnight stay in a hospital).

Document 3 covered 12-month postoperative outcomes including audiometrical results and postoperative complications. The occurrence of symptoms associated with chorda tympani lesions and newly experienced or intensified tinnitus and dizziness were recorded. Possible postoperative infections were recorded.

One year after surgery, a patient questionnaire was distributed. The following questions were evaluated:

- Are you satisfied with the preoperative counselling? (Score 1–5 where 1 is very satisfied and 5 is very dissatisfied).
- How do you experience your hearing 1 year after surgery? (Score 1–5 where 1 is much better and 5 is much worse).
- Do you reveal any new symptoms 1 year after surgery?

Space was left for the patients to add their own comments.

### Audiometry

Pure tone audiometry was performed not earlier than 1 month before the surgery and 12 months after the surgery. Air conduction (AC) included frequencies 0.25–8 kHz, and bone conduction (BC) included frequencies 0.5–4 kHz. The pure tone average (PTA_4_) used in the statistical analysis was based on the air conduction thresholds and bone conduction thresholds at frequencies 0.5,1, 2 and 4 kHz. The air-bone gap (ABG) was calculated by subtracting the BC from the AC for the same four frequencies (0.5–4 kHz). In cases of profound hearing loss/deafness with no measurable hearing thresholds, the AC was documented as 130 dB hearing level (HL), and the BC was documented as 75 dB HL.

We defined and used the parameters of successful surgery as (a) postoperative ABG ≤10 dB, (b) AC improvement ≥20 dB and (c) BC not worsened by ≥5 dB [6].

A questionnaire was sent to each participating clinic to investigate the number of surgeons performing stapes surgeries, the number performed by each surgeon and the amount of stapes surgery performed at each clinic per year.

### Statistical analysis

The data were expressed as the number of cases, percentages, means and standard deviations. Correlation between number of surgical procedures per clinic and audiological outcomes was tested using Fisher’s permutation test, within each individual.

## Results

In the present study, the results from patients undergoing surgery for primary otosclerosis between 2004 and 2010 were evaluated. Initially, 2493 registrations were collected from the SQOS but after repeated evaluation, 802 registrations with incomplete or missing audiograms were excluded. Three deaf ears were detected postoperatively and were not included in the analyses. Finally, 1688 registrations were evaluated.

At the introduction of the register in 2004, 31 clinics joined the SQOS. Due to discontinuation and fusion of clinics, 24 out of 26 active surgical clinics contributed during the study period. Only two clinics denied participation stating lack of spare time at the clinic. The number of registered surgical procedures in each clinic varied between 8 and 402 operations during 6 years. The questionnaire sent to the clinics regarding the number of stapes surgeries performed per surgeon revealed that 2 surgeons performed less than 5 cases, 4 surgeons 6–10 cases, 6 surgeons 11–15 cases, 3 surgeons 16–20 cases, 3 surgeons 21–25 cases, none 26–30 and 3 more than 31 cases.

Sixty-two per cent of the patients were female, and 38% were male. The mean age was 49.5 years (SD 12.1; range 15–90 years). Among the females, the mean age was 48.7 years (SD 12.1; range 15–90 years), and among males it was 50.5 years (SD12.1; range 15–85 years) shown in Table 1.

**Table 1:** Demographic characteristics of the otosclerosis cohort and surgical techniques used

Sex	Female	62%
Age	Years, mean (range)	49.5 years (15–90)
Surgical procedure	Stapedotomy	97.2%
	Stapedectomy	2.7%
Anaesthesia	Local	70%
	General	30%
Type of care	Overnight stay	65%
	Outpatient	35%

Stapedotomy was performed in 97.2% of cases, and stapedectomy in 2.7%. In the document for surgical report, no mention is given to intra-operative findings as a cause of the stapedectomies. Space is left for the surgeons to add their own comments.

Seventy percent of the surgical procedures were performed under local anaesthesia (Table 1). Treatment with an overnight stay in the hospital was more frequent (65%) compared with outpatient surgery (35%) (Table 1).

At the 1-year follow-up, the incidence of reported symptoms by the surgeon was overall low. The reported complication was tinnitus (4.7%), followed by chorda tympani symptoms (2.9%), dizziness (1.6%) and infections (0.7%).

Eighty-six per cent (1468/1688) of the patients responded to the patient questionnaire. Evaluation of the questionnaires revealed that a majority of the patients (92.7%) were satisfied and 0.4% was dissatisfied with the preoperative information. Closer examination revealed that the dissatisfied patients belonged to two different clinics, whom both had failed written information preoperatively.

Ninety-three percent (92.9%) of the patients reported improved hearing and 1.4% experienced a decline in hearing level shown in Table 2. A discrepancy between the incidence of reported complaints between surgeons and patients was detected. Post-operative complaints were noticed from surgeons in 9.4% (160/1688) versus 23% from patients (344/1468). Eighty-one patients reported tinnitus to the doctor and another 42 patients reported tinnitus only in the patient questionnaire. Symptoms addressed only by the patients included, i.e., sensitivity to sound, distortion of sound, a sensation of blocked ear, pain and reduced perception of touch.

**Table 2:** Subjective hearing experienced 1 year after surgery

Much better	63%
Better	29.9%
No difference	5.7%
Worse	1%
Much worse	0.4%

### Audiometry

All the audiometric results are presented in Table 3. The median preoperative PTA_4_ AC was 58 dB HL, and the BC was 19 dB HL. The ABG was 38 dB. 1 year after surgery, and the corresponding results were AC 26 dB HL, BC 17 dB HL and ABG 10 dB. The mean hearing gain after surgery, expressed as improvement of the AC, was 32 dB. Sixty-three per cent of the patients improved by more than 20 dB, and 89% improved by more than 10 dB. 1 year after surgery, the BC was maintained or not worsened by more than 5 dB in 93% of the patients. In 69%, the ABG was within ≤10 dB, and in 97% it was within 20 dB (Table 3). The 46 stapedectomies presented identical hearing results postoperatively compared to the stapedotomies, PTA_4_ AC 27 dB HL, BC 19 dB HLand ABG 8 dB.

**Table 3:** Pure tone audiometry AC, BC and ABG, mean values and standard deviations are presented, PTA_4_ (frequencies; 500, 1000, 2000 and 4000 Hz)

Pure tone audiometry-results								
Pre operative	500 Hz	1000 Hz	2000 Hz	3000 Hz	4000 Hz	6000 Hz	8000 Hz	PTA_4_
AC	58 ± 13.8	56 ± 15.1	52 ± 17.2	51 ± 19.7	54 ± 21.7	58 ± 22.7	58 ± 24.3	55 ± 14.9
BC	19 ± 12.2	23 ± 12.2	34 ± 14.2	30 ± 15.7	26 ± 17.4			25 ± 11.6
ABG	38 ± 12.7	34 ± 12.0	18 ± 11.3	21 ± 12.1	28 ± 13.4			29 ± 9.8
Post operative								
AC	26 ± 12.7	29 ± 12.6	31 ± 15.0	31 ± 17.2	38 ± 19.2	48 ± 21.8	55 ± 24.4	31 ± 12.5
BC	17 ± 11.2	19 ± 12.1	27 ± 14.5	25 ± 16.0	25 ± 17.8			22 ± 11.5
ABG	10 ± 9.1	9 ± 7.7	4 ± 6.8	6 ± 6.9	13 ± 9.5			9 ± 5.5

Successful surgery								
ABG		Improvement AC		BC worsened by				
≤10	69%	≤10 dB	11%	>5 dB	7%			
11–20	28%	11–20 dB	26%					
≥20	3%	21–30 dB	37%					
		>31 dB	26%					

Successful surgery measured as: ABG closure ≤10 dB, improvement in AC ≥ 20 dB and BC not worsened by ≥ 5 dB

**Fig. 1:**
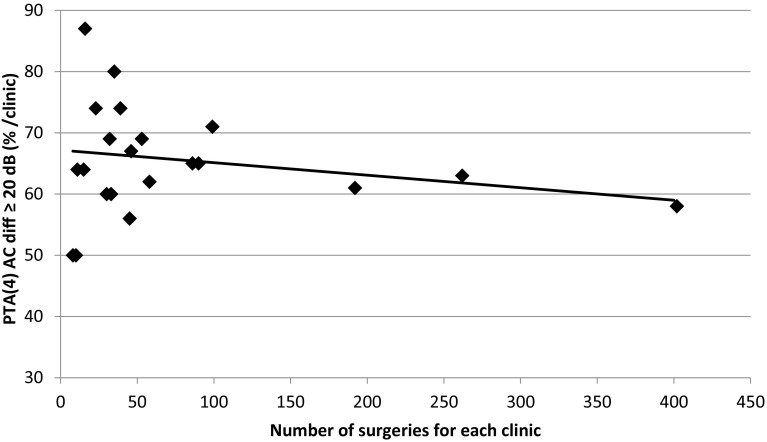
The percentage of successful surgeries in relation to number of performed surgeries per clinic are presented. Improvement in AC ≥ 20 dB. The linear regression model shown in Figs. 1, 2, 3, was performed with individual values of number of surgical procedures per clinic as independent variable and audiological outcomes as dependent variable.

**Fig. 2:**
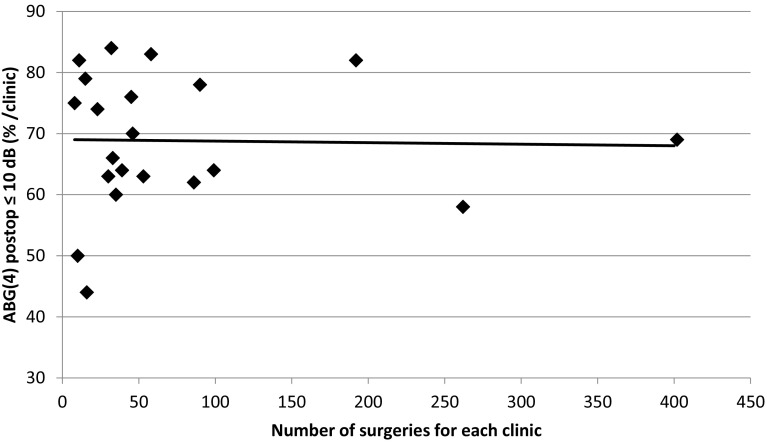
The percentage of successful surgeries in relation to number of performed surgeries per clinic are presented. ABG closure ≤10 dB

**Fig. 3:**
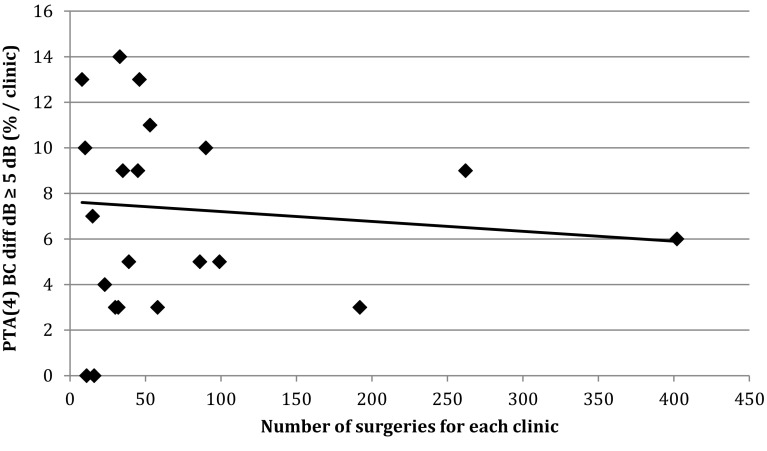
The percentage of successful surgeries in relation to number of performed surgeries per clinic are presented. BC not worsened by ≥ 5 dB

The percentage of individuals with postoperative ABG ≤10 dB, AC improvement ≥20 dB and BC not worsened by more than 5 dB was calculated for each participating ENT clinic, and the results are presented sequentially according to the number of patients included in the analyses in Figs. 1, 2 and 3. We found no correlation between the audiological outcomes and the number of surgical procedures performed in the present study with the exception of AC improvement ≥20 dB. Clinics performing a larger number of surgeries showed less improvement than clinics performing a smaller number of surgeries.

## Discussion

The SQOS covers more than 80% of the stapes surgeries annually performed in Sweden and offers a unique opportunity to study the quality indicators for this surgery. All the results are available for all the ENT clinics, and they are partially available to the public. To the best of our knowledge, comparable registers are not available in any other country.

Stapedotomy was the most common technique used, and stapedectomy was performed in only 2.8% of all cases. The use of local anaesthesia was noticeably more frequent than the use of general anaesthesia. Stapes surgery under local anaesthesia is generally considered safer due to the possibility to observe changes in the hearing and the appearance of dizziness, which facilitates the choice of an adequate length of the prostheses. Wegner et al. [7] observed no differences in the postoperative hearing outcomes when comparing patients undergoing stapes surgery under local anaesthesia compared with general anaesthesia. For medical reasons, stapes surgery can typically be accomplished in day-care units, which is preferable due to the reduced risks for postoperative infections and lower treatment costs [8]. A majority of the operations were carried out with overnight stay, which might be explained by long travel distances in a sparsely populated country like Sweden. In accordance with previous studies [9, 10], tinnitus and taste disorders were the most reported patient complaints, followed by dizziness. Evaluations and comparisons of the surgeon’s documentation and the patient questionnaires revealed discrepancies concerning “additional complaints”. This might be explained by the fact that the patients filled in the patient questionnaires at home independently of the surgeon. Another explanation might be that the patients have different expectations of the surgery. As emphasized in other studies [11], the evaluation and outcomes of the patient questionnaires are dependent on the preoperative information given and the expectations of the patients. Patients with eager expectations might be disappointed, and patients with lower expectations will communicate impressions that are too positive. This accentuates the importance of both oral and written preoperative information, which should include all the possible risks for complications, such as sound distortion and “blocked” ear. The preoperative information seems to be one of the most important quality indicators for surgical treatment [12, 13]. In compliance with these results, a national-based preoperative patient information program has been designed, which now is available online.

In accordance with other authors, more than 90% of the patients reported better hearing 1 year after surgery[10, 14]. Sixty-three per cent of the patients displayed a greater than 20 dB improvement of the AC, and the ABG was reduced to 10 dB or less in 69% of the cases. The improvement of the AC in the present study was comparable with a previous study [15], but it was poorer compared with earlier reported results by one single surgeon [16]. The audiological outcomes depend on which frequencies the PTA is based on. Generally, better results are achieved for PTA_0.5–3 kHz_ compared to PTA _0.5–0.4 kHz_, which has been shown in another study of a large series of one single surgeon [17]. Clinics with high amounts of surgery showed less percentage of AC ≥20 dB that could be explained by surgery performed on patients with less ABG, severe sensorineural hearing loss and cochlear implant candidates.

In 7% of the cases, the BC was worsened by more than 5 dB, but only 0.4% of the patients reported much worse hearing. This can be explained by a sufficient ABG-closure.

The quantity of stapes surgeries performed annually by each clinic in Sweden varied to a great extent. Only two clinics reported more than 80 operations annually. The audiological results of these two clinics were not specifically compared with the other clinics in this study. Yung et al. [18] showed that surgeons and clinics that reported larger numbers of stapes surgeries achieved better hearing results and fewer complications. We found no correlation between the audiological outcomes and the number of surgical procedures performed in the present study except for AC ≥20 dB. An explanation for this might be the fact that stapes surgery in Sweden generally is introduced late in the training of otosurgeons, and stapes surgery is performed by a very limited number of surgeons. In most clinics, only one or two surgeons perform the procedure which was confirmed by the questionnaire. Clinics performing less surgery per year often engage an experienced colleague from another clinic to perform the surgery. The evaluation of the outcomes of revision surgery, which generally is considered more difficult and challenging, might reveal larger differences. This is the subject of an ongoing study.

Only three deaf ears out of 1688 with complete registrations were observed after surgery resulting in an estimated frequency of less than 0.17%, which is comparable with similar international studies [5, 16, 19, 20]. A weakness in this register is that registration is not mandatory which can be one explanation to the few reports of deaf ears.

## Conclusion

The database might serve as a source for refinement and improvement of the surgical technique used for individual surgeons and institutions. In the open access of the homepage, patients can receive information concerning the number of surgeries performed per clinics, the national results of hearing outcome, complications and complaints. The SQOS provide a unique possibility to measure outcome and make improvement in health care.
